# Decreased thickness of the individually-mapped genital cortex after childhood sexual abuse exposure in adult women

**DOI:** 10.1038/s42003-026-09627-6

**Published:** 2026-02-19

**Authors:** Yuliya Kovalchuk, Sydney Schienbein, Andrea J. J. Knop, Martin Bauer, Stephanie Spengler, Michael Brecht, John-Dylan Haynes, Christine Heim

**Affiliations:** 1 Charité – Universitätsmedizin Berlin, corporate member of Freie Universität Berlin and Humboldt-Universität zu Berlin, Institute of Medical Psychology, Berlin, Germany; 2 Max Planck School of Cognition, Leipzig, Germany; 3 Charité – Universitätsmedizin Berlin, corporate member of Freie Universität Berlin and Humboldt-Universität zu Berlin, Experimental and Clinical Research Center, Berlin, Germany; 4 Bernstein Center for Computational Neuroscience, Berlin, Germany; 5 Humboldt-Universität zu Berlin, Department of Biology, Berlin, Germany; 6 NeuroCure Cluster of Excellence, Berlin, Germany; 7 Charité – Universitätsmedizin Berlin, corporate member of Freie Universität Berlin and Humboldt-Universität zu Berlin, Berlin Center for Advanced Neuroimaging, Berlin, Germany; 8 Humboldt-Universität zu Berlin, Department of Psychology, Berlin, Germany; 9 German Center for Mental Health, Berlin Potsdam Partner Site, Berlin, Germany

**Keywords:** Risk factors, Cortex

## Abstract

Previous research suggests interindividual variability in the location of the genital representation field and use-associated structural variation of genital field thickness associated with normative sexual activity in adult women. Using a sensory-tactile fMRI paradigm, we individually mapped genital fields of 128 women with and without exposure to childhood sexual abuse. We assessed whether structural variation of the individual genital field is driven by exposure to childhood sexual abuse or sexual frequency in the past year. We show that exposure to childhood sexual abuse associated with reduced thickness of individually-mapped genital cortex. Earlier abuse onset predicted greater reductions of genital field thickness. There was no effect of sexual frequency in the past year on genital field thickness. Classic neuroplasticity research indicates amplifying effects of stimulation on sensory cortex. In contrast, our results show long-lasting damaging effects of inappropriate stimulation during early development, emphasizing the need to protect children from sexual adversity.

## Abstract (web summary)

Structural mapping of the female genital representation in somatosensory cortex reveals experience-dependent plasticity and associations with childhood sexual abuse, and adult sexual behavior.

## Introduction

The developing brain demonstrates a remarkable capacity for neuroplastic reorganization in response to damage, environmental pressures, and experience^1^. Both animal and human studies have decisively shown that the brain is especially susceptible to modulation during so-called sensitive developmental periods^2^. The onset and duration of this circumscribed sensitivity depends on the area’s function, its connections, and the presence or absence of age-appropriate experience^2,3^. Sensory cortices are among the most vulnerable regions of the brain, as they develop early in life and remain relatively stable thereafter^4^. These areas serve as critical interfaces between the external world and internal experiences, potentially mediating how sensory experiences are encoded and perpetuated throughout the lifespan.

Under adverse conditions, such as early-life trauma or sensory deprivation, neuroplasticity facilitates compensatory changes that enable individuals to adapt their senses and survive^5,6^. However, later in life, these structural changes may underlie sensory deficits like chronic pain, decreased tactile acuity or hyper-vigilance^7–9^. Indeed, studies show that each distinct type of abuse exposure is associated with alterations in the specific sensory systems that convey the aversive experience, though evidence remains preliminary and overlapping gray matter alterations have been reported across modalities^10^. For instance, adults who witnessed domestic violence during childhood show reduced gray matter volume and cortical thickness in the visual cortices^11^. Similarly, adults who experienced verbal abuse as children exhibit structural alterations in brain regions associated with language processing^12^.

The impact of abuse involving touch on the primary somatosensory cortex (S1) is vastly understudied. It is conceivable that such forms of abuse may affect the representation fields of associated body parts in S1. Childhood sexual abuse (CSA) is an aberrant, developmentally inappropriate experience that is highly common and strongly associated with adverse outcomes, including sexual dysfunction and chronic genital or pelvic pain^13,14^. The genital representation field is likely particularly susceptible to neuroplastic effects of CSA. In support of this hypothesis, previous work demonstrated decreased cortical thickness of the putative genital representation field, predicted by the severity, onset age, and duration of abuse, suggesting an experience-associated neurodevelopmental effect that is opposite to the classical ‘Use it or Lose it principle’ of neuroplasticity, and may reflect sensory gating of aversive stimulation^15^. Importantly, however, the study did not individually localize the genital field for each woman and, therefore, these findings lack precision. Moreover, it remained unstudied whether this effect was indeed driven by CSA exposure or whether it may instead reflect reduced sexual activity in adulthood, commonly reported in CSA survivors, thus consistent with established principles of use-dependent neuroplasticity.

The genital representation field is a generally understudied area of S1 that plays a critical role in processing sensory input from the genitals. Animal models demonstrate that genital cortex shows a sexually monomorphic representation of genitalia with projections to various cortical and subcortical regions involved in sexual processing^16^. Unlike other parts of S1, the genital cortex undergoes late expansion during puberty and is modulated by sexual experiences^17^. In rats, direct physical contact and touch from sexually experienced adult males is a key driver of genital cortex expansion and accelerates female sexual maturation^18^. In humans, the precise location of genital cortex had been a matter of longstanding debate regarding the question as to whether the genital cortex represents an exception in the somatotopic organization, being located below the feet, or whether its location in S1 follows the expected topography of human body^19,20^. Our recent sensory-tactile functional mapping study provided compelling support that the genital field is located in the postcentral gyrus in somatotopic order^21^. We further demonstrated profound variability in the precise location of the genital field between women. Hence, for studies assessing structural variation of the genital field, it is necessary to localize the genital cortex at an individual level. By individually localizing the genital cortex, we demonstrated a correlation between genital field thickness and weekly sexual frequency in the past 12 months in healthy adult women. This finding suggests that under the normative circumstances the human and rodent genital cortex operate under the same principles of use-associated neuroplasticity.

Based on this new knowledge, we here use individual mapping to study the impact of CSA exposure on genital cortex with high precision: First, we provide converging evidence of the genital cortex location in a largest yet sample of women. Second, we employ a group-level analysis to examine whether the genital representation field is thinner in women with CSA exposure as compared to age-matched women with no such exposure. Third, we elucidate the contribution of CSA exposure versus adult sexual frequency to thickness of the genital cortex by correlating cortical thickness in the individually-mapped field with partnered weekly sexual activity, involving genital touch, in the past 12 months. This work not only resolves key questions about the human genital cortex but also substantiates our understanding of how CSA impacts sensory processing and cortical organization. Our findings hold potential to inform targeted interventions to mitigate the long-term impact of CSA on sexual health.

## Results

### Demographic and behavioral data

Demographic and behavioral characteristics of the sample are provided in Table 1. As expected, reflecting age-matching due to the established effect of age on cortical thickness, there was no significant difference in mean age between groups (CSA group: *M* = 31.6, *SD* = 7.38; non-CSA group: *M* = 30.6, *SD* = 7.04; *t*(125.72) = –0.80, *p* = 0.43). The majority of participants (93.7%) were of European descent, had higher education or were enrolled in university (81.2%), were of heterosexual orientation (85.1%), and were in a monogamous partnership (64%). There were no between-group differences in these features (Fisher’s exact test, all *p* > 0.05, two-sided).

**Table 1:** Demographic and Behavioral Characteristics of the Sample

	Control (n = 64)	CSA (n = 64)
Age, mean ± SD	30.6 ± 7.04	31.6^ns^ ± 7.38
Age of menarche, mean ± SD	13.1 ± 1.34	12.4^**^ ± 1.39
Ethnicity, n (%)		
European	63 (98.4%)	57 (89%)
Latin	-	4 (6.2%)
Middle Eastern	1 (1.6%)	-
South African	-	1 (1.6%)
American	-	1 (1.6%)
Asian	-	1 (1.6%)
Education, n (%)		
University degree (e.g., bachelor's, master’s, or equivalent)	34 (53%)	31 (48.4%)
Enrolled in university	21 (33.4%)	18 (28%)
Vocational school diploma	9 (13.6%)	10 (15.6%)
No formal vocational qualification	-	3 (4.8%)
No response	-	2 (3.2%)
Sexual Orientation, n (%)		
Heterosexual	54 (84%)	55 (86%)
Bisexual	4 (6%)	5 (7.6%)
Homosexual	3 (5.2%)	2 (3.2%)
Pansexual	1 (1.6%)	1 (1.6%)
No response	2 (3.2%)	1 (1.6%)
Partnership, n (%)		
Monogamous partnership	43 (67%)	39 (61%)
Polygamous partnership	-	2 (3%)
No partnership	21 (33%)	23 (36%)
Handedness, n (%)		
Right-handed	59 (91.6%)	54 (84%)
Left-handed	3 (5.2%)	3 (5.2%)
Ambidextrous	2 (3.2%)	7 (10.8%)
Hormonal Contraception, n (%)	17 (26.5%)	12 ^ns^ (19%)
Age of menarche, mean ± SD	13.1± 1.34	12.4**± 1.39
Sexually active in their lifetime, n (%)	62 (96.8%)	63 (98.4%)
Sexually active in the past year, n (%)	59 (91.6%)	51 (80%)
Sexual behavior, mean ± SD		
Frequency of sexual contact/week within the past 12 months	1.72 ± 1.5	1.0** ± 1.21
Age of first consensual sexual contact	16.3 ± 2.79	14.9^**^ ± 2.70
Lifetime number of sexual intercourse partners	15.5 ± 15.9	20.4^ns^ ± 25.6
Sexual satisfaction^1^	6.92 ± 2.35	5.02^***^ ± 2.83
Orgasm intensity^1^	7.77 ± 1.67	6.97^*^ ± 2.28
Ratings of Sensory-Tactile Clitoral Stimulation^2^, mean ± SD		
Intensity	4.14 ± 1.19	4.08^ns^ ± 1.26
Pleasantness	5.08 ± 0.93	4.23^***^± 1.29
Sexual arousal	3.84 ± 1.52	3.31^ns^ ± 1.65
Pain	1 ± 0^NaN^	1.5 ± 1.3

^1^1= very low till 10 = very high; ^2^7-point Likert scale: 1 = not at all, 4 = neutral, 7 = very much; ^ns^ = not significant, ^*^= p-value < 0.05, ^**^= p-value < 0.01, ^***^= p-value < 0.001, according to an appropriate t-test (Student’s or Welch’s), ^Nan^ – no variability.

Mean weekly frequency of sexual intercourse over the past 12 months was 1.0 times per week (*SD* = 1.21) in the CSA group as compared to 1.72 times per week (*SD* = 1.5) in the group with no CSA exposure. Women with a history of CSA reported significantly earlier mean age of menarche as compared to the women with no history of CSA (CSA group: *M* = 12.4, *SD* = 1.39; non-CSA group: *M* = 13.1, *SD* = 1.34; *t*(125.84) = 2.71, *p* = 0.007), as well as a significantly earlier age of first consensual sexual contact (CSA group: *M* = 14.9, *SD* = 2.7; non-CSA group: *M* = 16.3, *SD* = 2.79; *t*(122.7) = 2.78, *p* = 0.006). Mean intensity and arousal during the stimulation paradigm were rated as neutral. The stimulation was rated as non-aversive. There were no group differences in these parameters. Women with CSA exposure rated the stimulation as significantly less pleasurable as compared to non-exposed women (CSA group: *M* = 4.23, *SD* = 1.29; non-CSA group: *M* = 5.08, *SD* = 0.93); *t*(114.44) = 4.23, *p* = < 0.0001). Mean scores for both groups were in the neutral range.

### Individual Localization of the Human Female Genital Representation Field

**Fig. 1:**
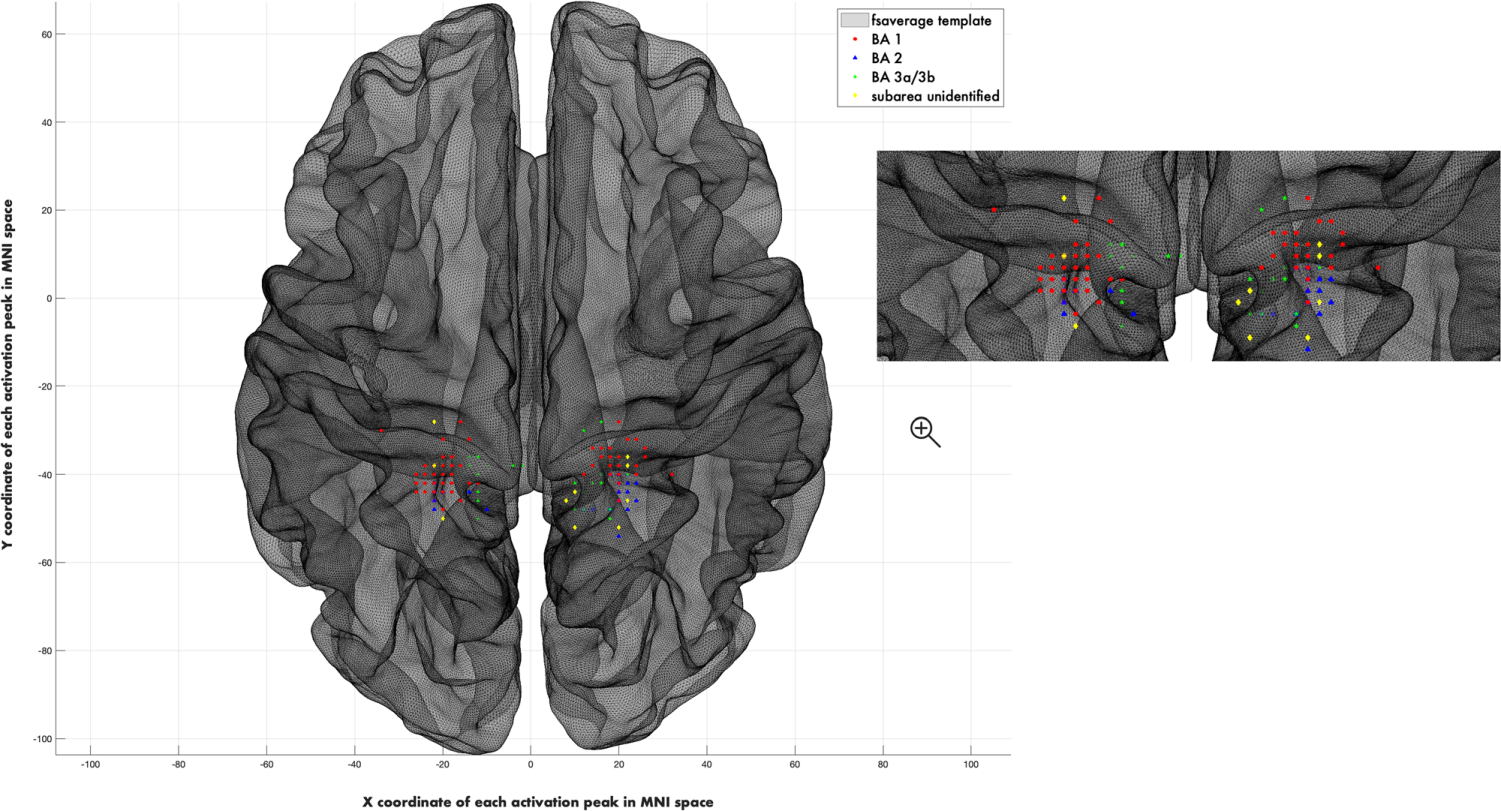
Individual mapping of genital cortex. Focal neural activations occurred in Brodmann areas 1, 2, and 3a/3b for all subjects. MNI barycentres of genital cortex in the left hemisphere [N = 89, x = −19 (SE: ± 5, range: -34 to -2), y = −38 (SE: ± 4, range: -50 to -28), z = 72 (SE: ± 7, range: 50-80)] and right hemisphere [N = 106; x = 19 (SE: ± 4, range: 8-32), y = -41 (SE: ± 5, range: -54 to -28), z = 71 (SE: ± 4, range: 62-82)].

The sensory-tactile stimulation paradigm reliably produced focal neural activations in S1 as measured by fMRI (Fig. 1). Individual localization of the left genital cortex was successfully achieved bilaterally in 86 participants. Additionally, five participants exhibited a left hemispheric activation only and 23 participants exhibited a right hemispheric activation only. One participant was excluded following outlier detection. One participant was excluded due to the activated cluster not reaching ten vertices. One participant was excluded due to inconsistent responses about abuse onset and the presence or absence of genital touch. The right index finger was successfully localized in 126 participants. Participants that exhibited activation in at least one hemisphere of the genital cortex and an activation in the right index finger area of S1 were kept for the subsequent analyses, resulting in n = 89 for left genital cortex and *n* = 106 for right genital cortex. For the right index finger, analyses were conducted separately for the subsets corresponding to the final genital cortex samples for the left and right hemispheres (*n* = 89 and *n* = 106) to ensure that any differences are not arising due to different participants being included in the analysis.

**Fig. 2:**
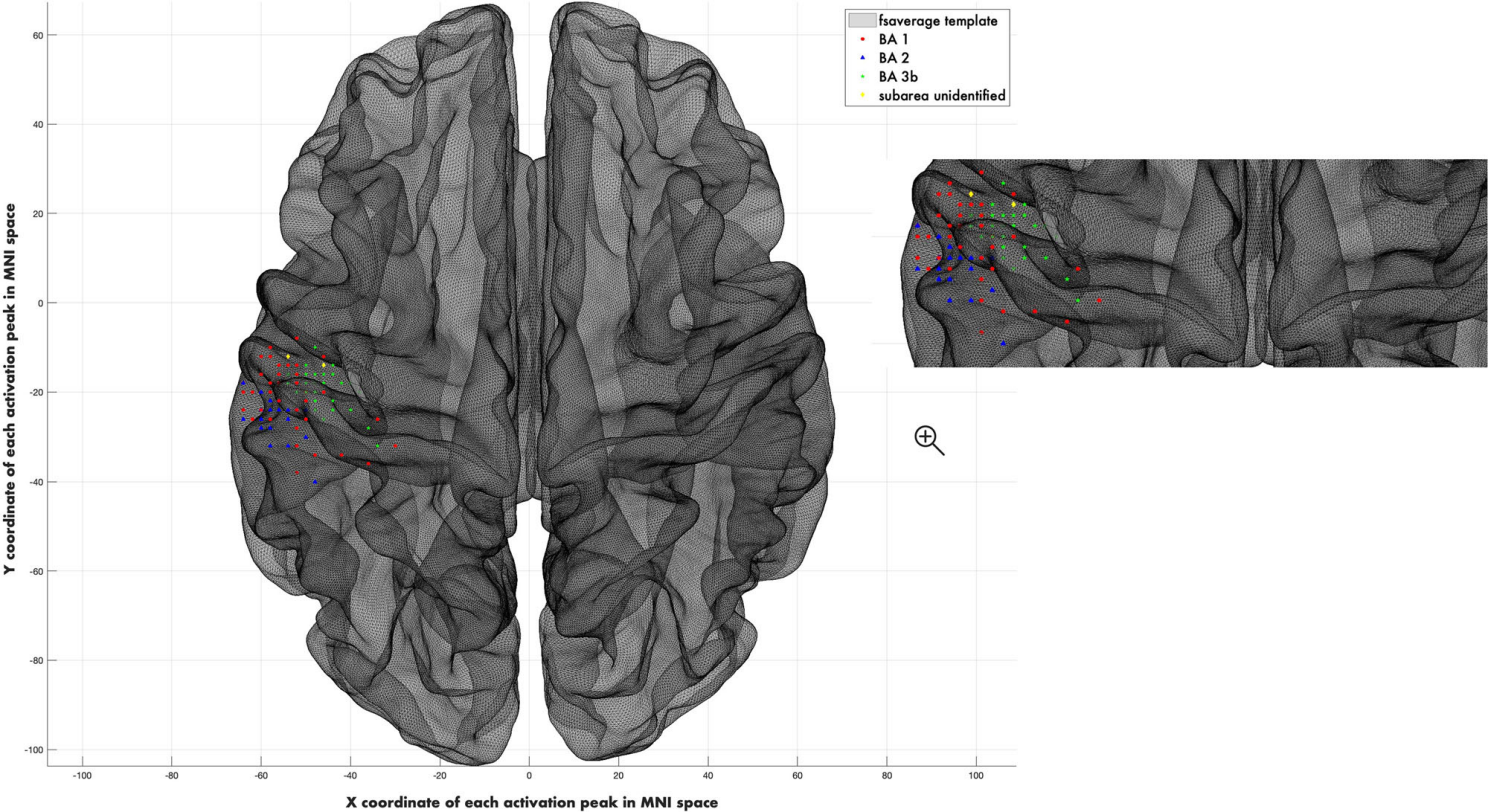
Individual mapping of right index finger cortex. Focal neural activations occurred in Brodmann areas 1, 2, and 3b for all subjects (N = 112). MNI barycentres [x = -51 (SE: ± 9, range: -69 to -10), y = − 21(SE: ± 6, range: -40 to -8), z = 53 (SE: ± 9, range: 36-84)].

Focal neural activation in S1 occurred in Brodmann areas 1, 2, and 3a/3b for all subjects (Figs. 1, 2). Notably, the success rate of the individual localization in the genital representation field almost did not differ between the groups (CSA: 86% success rate; non-CSA: 90% success rate). The location of the genital cortex and right index finger cortex was similar in all the subjects and followed the principle of somatotopy.

### Cortical Thickness of Human Female Genital Representation Field as a Function of Exposure to CSA

A one-way ANCOVA was conducted to examine the effect of CSA on cortical thickness in the genital representation field, controlling for age and whole-brain mean cortical thickness. Preliminary checks confirmed that assumptions of normality of residuals (Shapiro-Wilk test: left hemisphere, *p* = 0.04; right hemisphere, *p* = 0.26) and homogeneity of variances (Levene’s test: left hemisphere, *p* = 0.20; right hemisphere, *p* = 0.24) were met. Although the Shapiro-Wilk test was significant in the left hemisphere, Q-Q plot of the residuals looked approximately linear and allowed us to proceed. In the right hemisphere, the assumption of homogeneity of regression slopes was violated, as indicated by a significant interaction between age and group, *F* (1, 101) = 2.54, *p* = 0.01. To account for this, the age × group interaction term was included in the final ANCOVA model for the right hemisphere. Variance inflation check confirmed no multicollinearity between the predictors (range: 1–1.26).

Genital representation field thickness in the right hemisphere was significantly lower in the group of women exposed to CSA as compared to the group of women with no history of CSA (CSA group: *M* = 2.13 mm, *SD* = 0.2; non-CSA group: *M* = 2.24 mm, *SD* = 0.24; F(1, 101) = 6.77, *p*^*fdr-corrected*^ = .02) with a small-to-moderate effect size (partial η² = 0.06). There was no general influence of age on genital cortical thickness (*p* > 0.05). However, we observed a significant interaction effect between group and age (*F*(1, 101) = 6.44, *p*^*fdr-corrected*^ = .02, partial η² = 0.06) with a larger between-group difference in genital cortex thickness at a younger age.

**Fig. 3:**
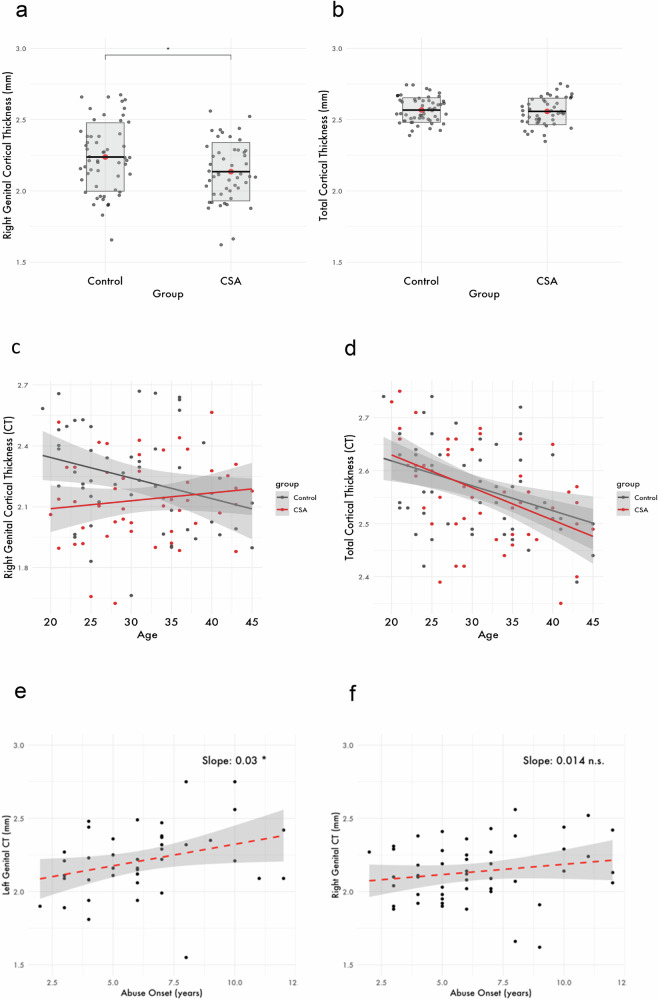
Effect of CSA exposure on genital cortical thickness. **a** Right genital cortical thickness as a function of group, F(1,101) = 5.72, *p* = 0.019, η² ≈ 0.0535. **b** Total cortical thickness as a function of group (*p* > 0.5). **c** Interaction of age and group in the right genital cortex, (*F*(1, 101) = 6.44, *p*^*fdr-corrected*^ = .02, partial η² = 0.06). **d**) Interaction of age and group in the whole-brain cortical thickness of the same subjects, (*p* > 0.5). **e** Left genital cortical thickness predicted by age of abuse onset in the group with childhood sexual abuse exposure, β = 0.03, SE = 0.01, t(40) = 2.15 p = 0.038. **f** Right genital cortical thickness predicted by age of abuse onset in the group with childhood sexual abuse exposure (*p* > 0.5).

In the left hemispheric genital cortex thickness, we observed a general association in the same direction, although it remained non-significant, (*F*(1, 85) = 1.42, *p*^*fdr-corrected*^ = .18). In the left hemisphere, there was a significant main effect of current age on genital field thickness (*F*(1, 85) = 5.23, *p*^*fdr-corrected*^ = .048, partial η² = 0.06) with lower genital cortical thickness at an older age. No group differences were observed in cortical thickness of the right index finger representation area or in whole-brain cortical thickness (all *p* > 0.05, FDR-corrected) in both hemispheres, underscoring the regional specificity of the genital field effect observed in the right hemisphere (Fig. 3).

Given that mean sexual frequency was significantly lower in the CSA group, we repeated the analyses with sexual frequency included as an additional covariate to determine whether the observed reduction in genital cortex thickness was attributable to CSA exposure or reduced adult sexual activity. The group difference in right genital cortex thickness remained significant, (*F* (1, 98) = 6.69, *p*^*fdr-corrected*^ = .02, partial η² = 0.06), suggesting that the effect is driven by an aversive age-inappropriate sensory experience in childhood rather than reduced sexual frequency in adulthood, in support of “developmental programming” hypothesis. The interaction between group and age became even stronger (*F* (1, 98) = 7.30, *p*^*fdr-corrected*^ = .01, partial η² = 0.07), suggesting that mild influence of cumulative sexual experience is still possible. In the left hemisphere, the results remained largely unchanged (group: *F*(1, 82) = 1.71, *p* = .19; age: *F*(1, 82) = 5.46, *p*^*dr-corrected*^ = .04; whole brain cortical-thickness: F(1, 82) = 24.81, *p*^*fdr-corrected*^ = . <.001).

To explore the “developmental programming” hypothesis further, we conducted an exploratory linear regression analysis to examine the relationship between the age at the onset of first CSA, as well as abuse duration in years, and structural thickness of the genital field. The first model revealed a statistically significant positive association between age at onset of CSA and structural thickness of the genital representation field in the left hemisphere (*β* = 0.03, *SE* = 0.01, *t*(40) = 2.17, *p* = 0.036). The overall model explained approximately 10.5% of the variance (*R²* = 0.24). This finding suggests that earlier abuse onset is associated with more pronounced decreases of thickness of the left genital field. Although this relationship was not statistically significant in the right hemisphere, the data has shown a similar direction of effect (*β* = 0.01, *SE* = 0.01, *t*(48) = 1.29, *p* = 0.2). No significant association was found between genital cortex thickness and abuse duration (*p* > 0.05).

### Structural Variation of Human Female Genital Representation Field as a Function of Past Year Sexual Frequency

We conducted partial correlation analyses to examine the association between mean weekly sexual frequency over the past 12 months and structural thickness of the individually-mapped genital representation field, controlling for current age, residualized years since onset of consensual sexual contact to account for cumulative sexual experience, and whole-brain cortical thickness. These analyses were performed separately for each hemisphere, first in the full sample and then stratified per group. Two additional participants were removed from this analysis because their mean weekly sexual frequency could not be established. No significant associations were found in the total group or either non-CSA or the CSA group (all *p*^*fdr-corrected*^ > 0.05). Thus, there was no evidence for structural variation in the genital cortex as a function of recent use.

## Discussion

We here provide new and robust evidence showing that the female genital representation field in S1 is remarkably modified by exposure to sexual abuse during early periods of brain development and, hence, we show that the genital cortex in humans has the capacity for reorganization in response to early-life experiences. Neuroplastic change after CSA consisting of decreased cortical thickness appears opposite to the well-accepted ‘Use-it-or-Lose-It’ principle of neuroplasticity. This neural adaptation appears to override use-dependent neuroplastic effects of adult sexual behavior frequency. Hence, our results support a ‘developmental programming effect’ that may serve to shield the child from an age-inappropriate aversive sensory experience. Such a developmental programming effect is further underscored by the fact that earlier abuse onset associated with decreased genital field thickness.

These results are relevant in several important ways: First, we consistently observe the location of the female genital representation field in the primary somatosensory cortex in somatotopic order, replicating our earlier finding in healthy young women^21^ in a much larger sample and for a broader age range. The original Penfield studies^22^, mapping sensory representation of men, localized the genital field in non-somatotopic order, deep in the medial wall, placing it next to the toes. The female somatosensory body map has, in general, been understudied^19^. Our study provides converging evidence of the somatotopic ordering of genital cortex, and this is highly consistent with the recent findings in both women and men that dispute the medial wall placement^23–25^. An important limitation of our study is that stimulation was confined to the external genitalia to ensure participant comfort and ethical compliance, thus precluding assessment of possible cortical representations of internal genital or viscero-sensory regions.

Second, we strengthen our previous finding of decreased cortical thickness of the somatosensory genital representation field as a function of exposure to CSA^15^, using a new group-level analysis and an individualized mapping approach. The average location identified through our individualized mapping is closely aligned with the putative location previously reported^15^. Using a higher methodological precision, we again observed that the cortical thickness of right genital representation field in CSA survivors was significantly lower than in the non-abused group and positively associated with the age of abuse onset. Of note, specific reductions in cortical thickness have also been observed in other sensory regions that correspond to the modality of abuse^11,12^. Since the developing brain is a highly plastic organ, it is especially sensitive to the organizing effects of lived experience^26^^.^ If an experience is highly aversive and developmentally inappropriate, the brain might narrow its cortical representation to limit detrimental effects, similar to “sensory gating” of abusive experiences^15^. While such plastic reorganization may serve a protective function during exposure to abuse, it could also constitute a neural substrate for maladaptive behavioral outcomes later in life, when typical behavioral expression would otherwise emerge, consistent with a ‘developmental programming’ hypothesis^15^. This would be in line with findings that survivors of CSA tend to have either compulsive or aversive attitude to engaging in sexual behavior in adulthood and experience a higher frequency of sexual dysfunction^27^. An alternative explanation would be that lower genital cortical thickness is a result of reduced frequency of current sexual intercourse in adulthood, which is one of the behavioral trajectories commonly reported in CSA survivors^27^. This would be in alignment with the “Use it or Lose it” principle, which posits that synaptic connections that are frequently activated are strengthened, while those that remain unused are gradually pruned^3^. In our study, we, for the first time, included weekly sexual frequency in the last year as a covariate of our model to assess the relative contribution of early-life versus adult experiences. We consider this a major contribution to the field. The group difference in genital cortical thickness was not affected by adding the sexual frequency to the model, and, importantly, we found no correlation between genital cortical thickness and sexual frequency in both groups. Therefore, our findings provide compelling support for the ‘developmental programming hypothesis’. While our findings are consistent with the hypothesis of early experience shaping cortical organization, they cannot establish developmental causality. Cross-sectional differences may reflect complex trajectories across development, including early enlargement followed by later atrophy. In our sample, we saw a larger difference in the genital cortex thickness as a result of CSA in younger participants, which seems to stay stable throughout the lifespan, while in the control group we observe an expected downward linear trend. One possible explanation could be that cumulative effects of sexual experiences and hormonal changes through life might override this group difference. Alternatively, it could be that the neuroplasticity in this area becomes limited. Unfortunately, our cross-sectional study design does not allow for a definitive interpretation. Future studies should employ longitudinal designs to probe into the possible trajectories of structural change in this area.

Third, our results failed to replicate the previously reported use-dependent structural variability in the female genital representation field as a function of recent normative sexual behavior in healthy young women with no CSA exposure^21^. Sexual behavior in the past year was not associated with genital field thickness in our study. Sexual behavior is highly variable across the lifespan, particularly in individuals without a stable partner. Chemical and neural signatures of sexual encounters may differ between early romantic, long-term and casual relationships^28,29^^.^ In comparison to the previous study, our sample exhibited considerable variability in the number of lifetime sexual partners, sexual preferences, and sexual activity within the past 12 months. It is possible that the chosen behavioral measure was not able to adequately capture the cumulative effect of these experiences. Future research employing longitudinal design with ecological momentary assessment of sexual behavior or experimental change in frequency is needed to scrutinize neuroplastic effects in genital cortex with normative sexual behavior.

Lastly, as a further substantiation of the ‘developmental programming hypothesis’, we were able to predict genital cortical thickness of left genital field by age of abuse onset, with lower cortical thickness values associated with an earlier onset age of the abuse. We did not find an effect of abuse duration. This suggests that there may exist a sensitive period and that aversive stimulation during this period induces neural effects that override effects of subsequent cumulative exposures throughout adulthood. Correspondingly, in the context of environmental enrichment, it has been reported that, in string players, age of onset of playing the instrument, but not cumulative practice time, predicted changes in the representation of the left hand in adulthood^30^.

Genital cortex thickness was significantly decreased only in the right hemisphere of women after CSA exposure, while the effect of age of abuse onset on genital cortex thickness was only observed in the left hemisphere. This suggests that there is lateralization in the effects of early-life sexual abuse. Previous studies on the genital cortex have primarily reported left-hemispheric effects^15,21^. Of note, structural alterations following early maltreatment have been observed in both left-hemisphere-only^10^ and right-hemisphere-only contexts^31^. In terms of sexual processing, left-hemispheric activation has been historically linked to sexual approach behaviors, while right-hemispheric activation is more commonly associated with sexual avoidance behaviors^32^. In a more general context, the right hemisphere has been associated with processing and expressing traumatic memories and negative emotions^33^. Although the possibility of structural lateralization is thought-provoking, especially in light of the emerging role of S1 in emotional regulation^34^, we cannot definitively confirm it based on our data. As our subset for the left hemisphere was smaller, it is possible that the true effects are bilateral, but we were not able to detect them due to insufficient statistical power. This limitation arose because our localization paradigm was not always successful and hence, did not achieve the necessary sample size, as indicated by the power analysis.

Future research is needed to scrutinize the mechanism underlying the observed genital cortex thickness decrease associated with early-life sexual abuse exposure. Several mechanisms may contribute, among them loss of neurons, decreased dendritic branching, spine density, synaptic connections, neurogenesis, and/or myelination within the cortical sheet^10,35^. A reduction in the number of neurons or impaired neurogenesis is unlikely, as sensorimotor areas develop early and tend to stay stable across the lifespan^4,36^. It is more plausible that existing neurons undergo functional alterations, such as changes in excitability, which could influence synaptic connectivity and dendritic branching patterns. Analogously, in rodent models, genital cortex expansion occurs through the invasion of thalamocortical afferent projections into layer four of the somatosensory cortex that reshape the cortical representation of genital sensory input, increasing the number of neurons that can be excited by genital stimuli, without altering their total number^18^. The aforementioned thalamus is a key relay station in the brain, acting as a “gateway” that transmits and filters behaviorally relevant sensory information to the cortex^37^. Aversive, age-inappropriate genital stimulation may lead to thalamic dysregulation, which could weaken or misdirect sensory input to the somatosensory cortex via bottom-up or top-down mechanisms. A bottom-up dysregulation could result from alterations at the lower levels of the sensory pathway - from the damage of peripheral receptors or weakened ascending afferents, leading to reduced efficacy in signal relay and synaptic transmission. This could manifest as a decrease in the number of dendritic spines on somatosensory cortical neurons, as has been observed when thalamic input is weakened or removed^38^. If the thalamus fails to deliver sufficient sensory input to the cortex, the corresponding cortical regions may further undergo synaptic pruning and volume reduction or the neuronal territories of body representations could reorganize, with genital field neurons potentially being taken over by neighboring regions. No studies to date confirm that CSA produces profound damage to the skin receptors in the genital area. It has been previously shown that poorer peripheral nerve functions and higher pain scores can be associated with lower connectivity of the thalamus and postcentral gyrus^39^. However, this more general effect of stress-related damage would not fully explain why the observed reduction in thickness is localized to the specific brain areas conveying a particular abuse type.

Alternatively, since the neocortex regulates its own thalamic input, it could potentially suppress overwhelming aversive stimuli through top-down control, akin to “sensory gating.” This intriguing hypothesis has been supported by studies that S1 modulates thalamic response to noxious stimuli^40^. It is also well-established that changes in mood or attention can induce top-down modulation, either enhancing or suppressing thalamic output and thus, changing sensory perception of incoming stimuli^41,42^. Notably, many women who have experienced sexual abuse develop attention-diverting coping strategies that can later become maladaptive, such as sexual dissociation and memory suppression^43^. Indeed, our sample of CSA survivors showed a higher incidence of sexual dissociation compared to the control group, which could in theory produce the top-down attentional modulation. Further investigations are needed to assess the integrity and functional connectivity of thalamocortical/corticothalamic circuits in women with a history of abuse to establish the direction of this relationship.

Additionally, it is worth considering the potential mechanisms that may modulate changes in cortical thickness. Sexual abuse during childhood can profoundly disrupt hormonal regulation via both the hypothalamic-pituitary-adrenal axis and the hypothalamic-pituitary-gonadal axis. CSA survivors often experience dysregulated cortisol levels, accelerated or delayed puberty, and/or hypogonadism^44^. Glutamatergic activity that plays a key role in mediating neural plasticity, particularly through NMDA receptor-dependent long-term potentiation and long-term depression is dysregulated by stress-related hormonal effects. This disruption may impair specific use-dependent plasticity, weakening the brain’s ability to adapt and reorganize in response to new sensory experiences^45^. Sex steroid hormones also induce well-known known fluctuations in cortical thickness and great matter volume across the menstrual cycle and in times of major hormonal change and may have an impact on genital field thickness^46,47^. In rodents, genital cortex expansion with the onset of puberty is mediated through estrogen^18^. Correspondingly, in humans, gray matter volume shrinkage in the putative right genital representation field of S1 has been observed in perimenopausal females and is associated with estrogen decline^48^. Future studies should incorporate hormonal assessments to better understand the potential influence of hormones on the genital field. As our sample included only participants assigned and self-identified as female at birth, the findings should not be generalized to genital cortex organization across sexes or gender identities. Future studies including male, transgender and intersex populations are needed to test whether similar experience-dependent plasticity patterns emerge.

Finally, it is crucial to understand the posited functional and behavioral implications of lower cortical thickness in the genital representation area of S1. Decreases in the cortical thickness of the somatosensory cortex are generally linked to reduced sensitivity and lower pain threshold^49,50^. Additionally, sensory integration may be disrupted, which could impair the sense of body ownership^51^. These changes in sensory processing may drive maladaptive sexual behaviors, such as the development of either aversion or sensation-seeking tendencies, as well as sexual dissociation.

In conclusion, neuroscientific evidence is essential for understanding the root causes of sexual dysfunction and the maladaptive behavioral strategies that survivors of sexual abuse may develop in adulthood^52^^.^ Our results underscore that these consequences may not be entirely within voluntary control, indicating that early trauma can exert profound and lasting effects on brain function and behavior. If sexual dysfunction is at least partly linked to structural alterations in the genital representation field, there is potential for inducing neuroplasticity in this area through targeted stimulation. One promising approach is training-independent sensory learning (TISL), which involves designing specific passive sensory stimulation protocols that precisely control the timing and spatial distribution of the stimulation. Such protocols can reshape brain organization, influencing both sensory perception and behavior. For example, applying TISL to the index finger has been shown to increase the size of cortical representation maps and improve tactile acuity within a short time span^53^. Intriguingly, improved tactile perception has been observed not only in the stimulated index finger but also in neighboring unstimulated body areas, such as the cheek and upper lip, suggesting that adjacent cortical fields may also be responsive to the stimulation^53^. This offers exciting possibilities for expanding the scope of targeted interventions for sexual dysfunction without direct genital stimulation. This perspective offers hope that our result will inform a development of novel approaches that address the neurobiological component of sexual functioning, ultimately enhancing treatment outcomes and providing more effective support for survivors.

## Methods

### Study Design

The study uses a cross-sectional design comparing structural thickness of the individually-mapped genital field between two groups of women with and without exposure to CSA. Features of CSA as well as frequency of normative sexual behavior in the past year were further correlated with genital field thickness within and across groups. The study was preregistered on the Open Science Framework^54^ and its hypotheses, methods, and analysis plan were specified prior to data analysis. The study was approved by the ethics committee of Charité—Universitätsmedizin Berlin and all regulations relevant to human research participants were followed. Participants provided written informed consent and received small monetary compensation for their time and effort.

### Sample

We recruited 128 healthy women aged 18 to 50 years, including 64 women with exposure to CSA and 64 women without such exposure from the general population via online and offline flyers and ads. Participants were age-matched on a group level to ensure similar mean age, as age has a proven effect on cortical thickness. For this study, CSA was defined as unwanted sexual experience that occurred before the onset of puberty, i.e.,menarche. Given that we aimed to assess neuroplastic effects of CSA on the genital somatosensory representation field, we limited the definition of CSA in this study to experiences that included genital touch. Sample size was determined using a priori power analysis in G*Power 3.1. Based on the effect sizes observed in Heim et al. (2013), the current sample size of *N* = 128 was determined using the formula R² = (F / (F + dfₑᵣᵣₒᵣ)) × (1/n) for an ANCOVA with two groups and two covariates to achieve 80% power at an α-level of 0.05.

Exclusion criteria included current psychotic or substance use disorders, neurological disorders, chronic physical disease, past brain or urogenital surgery, psychotropic substances taken within the past three months, current pregnancy or pregnancy in the past two years, current breastfeeding, current state of crisis, perimenopause or menopause and MRI contraindications. For the group not exposed to CSA, we excluded women who had experienced moderate to severe emotional or physical abuse or any sexual abuse based on the German version of the Childhood Trauma Questionnaire (CTQ)^55^. Exclusionary conditions were assessed using standard clinician-administered interviews and questionnaires^56^.

### Procedure and Assessments

Participants completed two visits at Charité—Universitätsmedizin Berlin. During the first visit, participants provided information on their demographic characteristic, as well as current and past physical and psychiatric health history. Experiences of childhood maltreatment, including sexual abuse, were assessed using the German version of the CTQ^55^. We further conducted a clinician-administered interview, i.e., the extended German version of the Maltreatment and Abuse Chronology of Exposure (MACE)^57^. Based on this information, women were classified into those with and those without exposure to CSA. Features of CSA, i.e. onset age and duration, were extracted from responses in the MACE. During the second study visit, participants underwent a functional mapping paradigm as well as structural MRI. Participants further completed a brief study-specific self-report questionnaire assessing sexual history, including frequency of normative sexual intercourse in the past year. Given the descriptive focus of these measures and the lack of validated tools capturing them, a custom questionnaire was appropriate and sufficient for the study’s aims.

### Sensory-Tactile Functional Mapping Paradigm

**Fig. 4:**
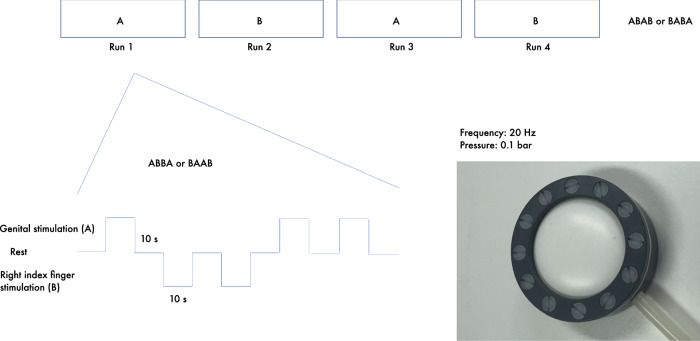
Sensory-tactile paradigm. Stimulation was delivered via an air-controlled oscillating membrane operating at a frequency of 20 Hz and pressure of 0.1 bar. The experimental paradigm followed an ABAB versus BABA block design, alternating between stimulation of the clitoral region (**A**) and the right index finger (**B**), with 10-second intervals of rest between stimulation phases. Each of the four fMRI runs began with a no-stimulation period and included a total of eight clitoral and eight index finger stimulation phases, presented in a counterbalanced order.

To individually localize the genital representation field in S1, we used a sensory-tactile functional MRI paradigm as previously described^21^ (Fig. 4) The functional MRI paradigm included four runs of a sensory-tactile stimulation of the clitoral region versus the right index finger. The paradigm was developed to be non-invasive, non-aversive, and non-arousing to ensure maximal comfort and to meet the utmost ethical standards. Women were asked to self-place a membrane on the clitoral region above standardized disposable underwear with no skin contact. The stimulation was delivered via air puffs producing short low-intensity pulses of the membrane. Sensory-tactile stimulation of the right index finger was used as a control condition. To minimize irrelevant brain activation, women were asked to close their eyes during the stimulation phase. To monitor potential dislocation of the membrane during the procedure, women were asked to circle the placement of the membrane on an anatomical image of the genital region after the scan. For quality control purposes, women were further asked to rate the pleasantness versus aversion, intensity, pain and potential sexual arousal during the clitoral stimulation as well as pleasantness versus aversion, pain and intensity of the right finger stimulation on a 7-point Likert scale.

### MRI Acquisition

Structural and functional MRI were obtained using a 3.0 T Siemens Magnetom Prisma fit scanner (Siemens Medical System) with a standard 64-channel head coil. One 0.8 mm3 isotropic T1-weighted anatomical scan was acquired using the magnetization-prepared rapid gradient echo sequence (MPRAGE) with a duration of 6:03 min and the following parameters TE  =  2.22 ms, TR  =  2400 ms, flip angle = 8, bandwidth = 220 Hz/Px, FOV  =  256 mm. Functional MRI scans were obtained using a T2*-weighted echoplanar image (EPI) pulse sequence (TR/TE = 2000/30 ms, slice number = 32, voxel size = 2 × 2 × 2 mm3, slice gap = 0.75 mm). The functional MRI paradigm consisted of four blocks with a duration of 5:36 min each. Details on scanning parameters are available in the preregistered protocol^54^.

### Anatomical and Functional Preprocessing

Anatomical preprocessing was carried out using the CAT12.9 (r2560) toolbox within SPM12, running on MATLAB_R2023b, with bias-corrected T1-weighted images. A quantitative quality metric was generated for each image, and only images rated as “satisfactory” or higher (IQR > 70%) were included, following the recommendations^58^. Functional preprocessing was performed using standard SPM12 functions. The first five “dummy” volumes were discarded to allow for T1 saturation. Spin-echo field maps were acquired for distortion correction. To improve primary motion correction accuracy, double-pass realignment with default value and 4th-degree interpolation was applied. Intrasubject registration to the mean and anatomical image was separately performed for each of the four scanning blocks using default co-registration settings with anatomical image as a reference image and the mean image as a source image. Data was high-pass filtered with a default cutoff period of 128 s to correct for slow drift artifacts. Spatial normalization to a standard stereotaxic space and image smoothing were omitted to enable subsequent cortical thickness analyses in native space. Functional images were assessed for motion parameters, and any images with head motion exceeding 2.0 mm of translation or 3.0° of rotation in any direction within a scanning block were excluded. The newly applied, more stringent criterion was chosen to match the size of our voxel resolution, and does not match our preregistration protocol.

### Individual First-Level Modeling

Functional MRI data was analyzed using a general linear model (GLM) with two within-subject conditions of interest (10 s of either clitoral or right index finger stimulation alternating with 10 s of rest) modeled using a boxcar function convolved with a canonical hemodynamic response function (HRF) and thresholded at *p* < 0.001 without correction or *p* < 0.05 with FWE-correction for multiple comparisons. For further details, refer to Knop et al. (2022)^21^. To localize the individual genital field, we saved the first most activated cluster in S1 as a region of interest. We then used the 10 most activated vertices identified through the above-described functional mapping paradigm to extract cortical thickness values for the further analysis.

### Assessment of Structural Thickness of the Individually-Mapped Genital Field

The CAT12 toolbox for SPM12 was used to perform cortical surface-based morphometry (SBM) of the anatomical images. The individually defined ROIs obtained through the first-level fMRI analysis for the bilateral genital representation area and right index finger representation area of S1 were separately mapped onto individual native space cortical surfaces of the left and right hemisphere. After cortical surface registration and resampling without smoothing, mean thickness of the ten functionally most active vertices within the individually mapped ROIs were calculated for each subject as part of central surface estimation^59^. As our stimulation was not individually calibrated, some clusters did not reach ten voxels or encompassed areas outside of the somatosensory cortex. Therefore, as an additional quality control step, we backprojected the ten most activated vertices onto a brain mesh and only included the 10 most activated vertices in S1.

### Statistics and Reproducibility

As a first step, we explored demographic and behavioral data by comparing groups on key variables, including age, mean weekly sexual frequency, paradigm ratings, age at menarche, and age at first consensual sexual contact, using independent-samples *t*-tests (Table 1). Categorical group differences were assessed using Fisher’s exact test (two-tailed), since in each case expected cell counts were below 5 for some categories.

For the first aim of the study, we conducted a one-way ANCOVA, covarying for current age and mean cortical thickness to compare structural thickness of the individually-mapped genital field between women with and without exposure to CSA. Given that, according to the recent reports, cortical thickness is largely independent of head size^60^, whole-brain mean cortical thickness rather than total intracranial volume (TIV) was included as a global covariate in all models, contrary to our preregistration protocol. To confirm the specificity of this effect, we conducted the same ANCOVA to compare structural thickness of the individually-mapped right index finger representation area, as well as for whole-brain cortical thickness. Subjects with thickness values exceeding three standard deviations above or below the mean were excluded as outliers.

For the second aim of the study, we conducted a partial correlation analysis to test the association between structural thickness of the individually-mapped genital representation field for each hemisphere and the mean sexual frequency per week in the last 12 months. Mean weekly sexual frequency was first calculated for each month, then added and divided by 12. We used current age, residualized years since first consensual sexual contact and whole-brain cortical thickness as covariates. These analyses were conducted both in the total sample of women and separately within each group. To confirm region-specificity, we conducted a partial correlation analysis to assess associations between left structural thickness of the right index finger representation in S1 and sexual frequency in the past year.

Lastly, given that we observed a significantly lower average sexual frequency in the past year in the group of women with exposure to CSA as compared to women without exposure to CSA, we repeated the between-group ANCOVA and included sexual frequency in the past year as a covariate. This analysis served to assess whether potential reduced genital field thickness is associated to the early-life aversive exposure (‘developmental programming’) versus a current behavioral change (‘Use-it-or-Lose-it principle’). To clarify this point further, we conducted an exploratory linear regression in the CSA group with age of sexual abuse onset and mean weekly sexual frequency in the first model, and duration of sexual abuse and weekly sexual frequency in the second model. The models were run separately, as age of abuse onset and its duration were correlated, to avoid the potential issue of collinearity. Given the exploratory nature of these analyses and the limited number of comparisons (left and right hemisphere), we did not apply FDR-correction for multiple comparisons in this case. Our aim was to identify potential lateralized effects that may guide future hypothesis-driven work. These findings should be interpreted cautiously and considered preliminary until replicated in independent samples.

All statistical analyses were computed using R Project for Statistical Computing (RStudio 2023.12.0 Build 369) and are available in our preregistration protocol^54^. Statistical significance was set at *p* < 0.05 with FDR-correction applied to all confirmatory but not exploratory analyses.

### Reporting summary

Further information on research design is available in the Nature Portfolio Reporting Summary linked to this article.

## Supplementary information

Reporting Summary

## Data Availability

The raw neuroimaging datasets and behavioral data generated and/or analyzed during the current study are not publicly available due to our data privacy agreement and ethical restrictions that serve the right of our participants to remain anonymous and protect them from potential identification. Source data to support the findings of this study are available as a part of OSF preregistration^54^.

## Code availability

Custom MATLAB Code (Version R2023b, MathWorks Inc.) for SPM12 and CAT 12, as well as custom code for R Project for Statistical Computing (RStudio 2023.12.0 Build 369) is available as a part of OSF preregistration^54^.
