# Supplementary material for: Decreased thickness of the individually-mapped genital cortex after childhood sexual abuse exposure in adult women
Source: Commun Biol. 2026 Feb 19;9:375. doi: 10.1038/s42003-026-09627-6 (PMC12992894; doi:10.1038/s42003-026-09627-6)
Supplement: Supplementary file 1 — Reporting Summary [file 42003_2026_9627_MOESM1_ESM.pdf]

nature portfolio

Corresponding author(s): Christine Heim, Ph.D.

Last updated by author(s): 2026 - 01-19

Reporting Summary

Nature Portfolio wishes to improve the reproducibility of the work that we publish. This form provides structure for consistency and transparency in reporting. For further information on Nature Portfolio policies, see our Editorial Policies and the Editorial Policy Checklist.

Statistics

For all statistical analyses, confirm that the following items are present in the figure legend, table legend, main text, or Methods section.

n/a	Confirmed	
☐	☒	The exact sample size (n) for each experimental group/condition, given as a discrete number and unit of measurement
☐	☒	A statement on whether measurements were taken from distinct samples or whether the same sample was measured repeatedly
☐	☒	The statistical test(s) used AND whether they are one- or two-sided<br>*Only common tests should be described solely by name; describe more complex techniques in the Methods section.*
☐	☒	A description of all covariates tested
☐	☒	A description of any assumptions or corrections, such as tests of normality and adjustment for multiple comparisons
☐	☒	A full description of the statistical parameters including central tendency (e.g. means) or other basic estimates (e.g. regression coefficient) AND variation (e.g. standard deviation) or associated estimates of uncertainty (e.g. confidence intervals)
☐	☒	For null hypothesis testing, the test statistic (e.g. F, t, r) with confidence intervals, effect sizes, degrees of freedom and P value noted<br>*Give P values as exact values whenever suitable.*
☒	☐	For Bayesian analysis, information on the choice of priors and Markov chain Monte Carlo settings
☒	☐	For hierarchical and complex designs, identification of the appropriate level for tests and full reporting of outcomes
☐	☒	Estimates of effect sizes (e.g. Cohen's d, Pearson's r), indicating how they were calculated

Our web collection on statistics for biologists contains articles on many of the points above.

Software and code

Policy information about availability of computer code

Data collection	Data for each participant was recorded/transferred using the Research Electronic Data Capture (REDCap) software, as well as standard paper-based demographic/clinical questionnaires. All MRI data were organized according to the BIDS standard and saved on internal servers.
Data analysis	All neuroimaging data was analyzed in SPM12 and CAT12, using custom MATLAB Code (Version R2023b, MathWorks Inc.). All statistical analyses were carried out in R Project for Statistical Computing (RStudio 2023.12.0 Build 369). All code will be provided upon reasonable request for non-commercial use.

For manuscripts utilizing custom algorithms or software that are central to the research but not yet described in published literature, software must be made available to editors and reviewers. We strongly encourage code deposition in a community repository (e.g. GitHub). See the Nature Portfolio guidelines for submitting code & software for further information.

Data

Policy information about availability of data

All manuscripts must include a data availability statement. This statement should provide the following information, where applicable:

- Accession codes, unique identifiers, or web links for publicly available datasets
- A description of any restrictions on data availability
- For clinical datasets or third party data, please ensure that the statement adheres to our policy

The individual-level raw neuroimaging datasets and behavioral data generated and/or analyzed during the current study are not publicly available due to our data

privacy agreement and ethical restrictions that serve the right of our participants to remain anonymous and protect them from potential identification. Group-level data, preprocessing data, as well as de-identified source data to support the findings of this study are available from the corresponding author on reasonable request. All requests will be handled within four weeks.

Research involving human participants, their data, or biological material

Policy information about studies with human participants or human data. See also policy information about sex, gender (identity/presentation), and sexual orientation and race, ethnicity and racism.

Reporting on sex and gender	All participants were assigned female at birth (XX chromosomes, presence of ovaries) and self-identified as female, in line with our sex-specific hypothesis. All gender identities were eligible for inclusion, provided participants had not initiated hormonal treatment or undergone gender-affirming surgery. Data on divergence between sex assigned at birth and gender identity was not collected in this study.
Reporting on race, ethnicity, or other socially relevant groupings	We did not include information on race/ethnicity in statistical analyses because we had no theory-driven hypothesis that ethnicity would influence the association between genital cortex thickness and childhood sexual abuse exposure and because vast majority of our participants self-identified as White/Causasian 120 people in the final sample were of European, 4 of Latin, 1 of Middle Eastern, 1 of American 1 of South African and 1 of Asian descent. Childhood sexual abuse was self-reported and defined as sexual abuse before the first menarche that included genital touch and could be clearly remembered.
Population characteristics	The final sample that complied with our inclusion criteria comprised of 128 women assigned female at birth (mean age: 31.13, SD: 7.20). 64 women self-reported exposure to childhood sexual abuse.
Recruitment	Participants were recruited from the general population of the metropolitan are of Berlin, Germany via online and offline ads and flyers, and previous studies at Charité -Universitätsmedizin Berlin. For both groups we recruited healthy women aged 18-50 years with no menopause symptoms. Exclusion criteria were current psychotic or substance use disorders, neurological disorders, chronic physical disease, past brain or urogenital surgery, psychotropic substances taken within the last three months, current pregnancy or breastfeeding, current state of crisis, and MRI-contraindications. For women without CSA exposure, we additionally excluded women who have experienced moderate to severe emotional or physical abuse or any sexual abuse.
Ethics oversight	The study was approved by the ethics committee of Charité -Universitätsmedizin Berlin and it adheres to the Declaration of Helsinki. Participants provided written informed consent and received small monetary compensation for their time and effort. To further establish that the participation was not financially motivated, participants were informed that they can cease participation without an explanation and the full amount will still be paid out to them.

Note that full information on the approval of the study protocol must also be provided in the manuscript.

Field-specific reporting

Please select the one below that is the best fit for your research. If you are not sure, read the appropriate sections before making your selection.

☐ Life sciences ☒ Behavioural & social sciences ☐ Ecological, evolutionary & environmental sciences

For a reference copy of the document with all sections, see nature.com/documents/nr-reporting-summary-flat.pdf

Behavioural & social sciences study design

All studies must disclose on these points even when the disclosure is negative.

Study description	Cross-sectional retrospective cohort study design comparing two groups of women with and without prior CSA experience.
Research sample	The final sample that complied with our inclusion criteria comprised of 128 women living in the metropolitan area of Berlin, Germany assigned female at birth (mean age: 31.13, SD: 7.20). 64 women self-reported exposure to childhood sexual abuse, 64 women confirmed not having such exposure.
Sampling strategy	Participants were recruited in the metropolitan area of Berlin, Germany, via online and offline message boards and flyers. Sample size was based on power analysis. The effect size was calculated based on Heim et al. (2013) with a formula R2 = (F/(F + dferror)) X (1/n). Using G Power 3.1 (Faul et al., 2007), we determined that for an ANCOVA with 2 groups and 2 covariates, a sample size of 128 participants (64 per group) is needed to achieve 80% power. Data collection was closed, when the target sample size was reached.
Data collection	The data were collected as part of a larger study that included additional neuroimaging and clinical assessments in the same participant sample. The study consisted of a prescreening call and two timepoints. At the first timepoint, clinical assessments were conducted in person by a trained clinician at the Institute of Medical Psychology, Charité – Universitätsmedizin Berlin. Demographic and clinical data were collected using standardized paper-based questionnaires. Information regarding abuse history was entered by participants via the Research Electronic Data Capture (REDCap) platform. At the second timepoint, MRI scanning was conducted at the Berlin Center for Advanced Neuroimaging (BCAN). Scanning sessions were always conducted in the presence of at least two staff members. For participants with a history of childhood sexual abuse, a trained therapist was present during scanning to provide immediate support in case of any distress. Following the MRI session, behavioral data related to sexual behavior were collected via REDCap. Additional data on sexual dysfunction (not relevant to the current analysis) were obtained through a clinician-administered

	interview. All participants who completed the optional satisfaction rating indicated that they were satisfied with the procedures and manner in which data were collected.
Timing	Data were collected from March 2023 until October 2024
Data exclusions	A total of N = 352 participants were identified. N = 243 were invited to a prescreening call (see below). N = 86 did not fulfill our inclusion criteria. N = 167 completed the T1 appointment - a clinical interview. N = 30 were additionally excluded due to not fulfilling our exclusion criteria based on clinical data or dropped out. N = 137 were invited to a T2 appointment - MRI scanning. N = 9 dropped out die to feeling claustrophobic in the scanner and not being able to proceed with the scan. Our final sample calculated by power analysis included 128 participants. Individual mapping was successful in Individual localization of the genital cortex was successfully achieved bilaterally in 86 participants. Additionally, five participants exhibited a left hemispheric activation only and 23 participants exhibited a right hemispheric activation only. The right index finger was successfully localized in 126 participants. One participant was excluded following outlier detection. One participant was excluded due to the activated cluster not reaching ten vertices. One participant was excluded due to inconsistent responses about abuse. Participants that exhibited activation in at least one hemisphere of the genital cortex and an activation in the right index finger area of S1 were kept for the subsequent analyses, resulting in n= 89 for left genital cortex and n = 106 for right genital cortex. For the right index finger, analyses were conducted separately for the subsets corresponding to the final genital cortex samples for the left and right hemispheres (n = 89 and n = 106) to ensure that any differences are not arising due to different participants being included in the analysis. Two additional participants were removed from the analysis of structural variation of the genital cortex as a function of past sexual frequency because their mean weekly sexual frequency could not be established.
Non-participation	A total of N = 352 participants were identified, N = 99 expressed no further interest, mainly due to time unavailability. Thus, N = 243 were invited to a prescreening call.
Randomization	Observational Study - Data was collected from study subjects that are not randomly assigned to a treatment.

Reporting for specific materials, systems and methods

We require information from authors about some types of materials, experimental systems and methods used in many studies. Here, indicate whether each material, system or method listed is relevant to your study. If you are not sure if a list item applies to your research, read the appropriate section before selecting a response.

Materials & experimental systems

n/a	Involved in the study
☒	☐ Antibodies
☒	☐ Eukaryotic cell lines
☒	☐ Palaeontology and archaeology
☒	☐ Animals and other organisms
☒	☐ Clinical data
☒	☐ Dual use research of concern
☒	☐ Plants

Methods

n/a	Involved in the study
☒	☐ ChIP-seq
☒	☐ Flow cytometry
☐	☒ MRI-based neuroimaging

Plants

Seed stocks	*Report on the source of all seed stocks or other plant material used. If applicable, state the seed stock centre and catalogue number. If plant specimens were collected from the field, describe the collection location, date and sampling procedures.*
Novel plant genotypes	*Describe the methods by which all novel plant genotypes were produced. This includes those generated by transgenic approaches, gene editing, chemical/radiation-based mutagenesis and hybridization. For transgenic lines, describe the transformation method, the number of independent lines analyzed and the generation upon which experiments were performed. For gene-edited lines, describe the editor used, the endogenous sequence targeted for editing, the targeting guide RNA sequence (if applicable) and how the editor was applied.*
Authentication	*Describe any authentication procedures for each seed stock used or novel genotype generated. Describe any experiments used to assess the effect of a mutation and, where applicable, how potential secondary effects (e.g. second site T-DNA insertions, mosiacism, off-target gene editing) were examined.*

Magnetic resonance imaging

Experimental design

Design type	Task (stimulation vs. rest), block design
Design specifications	The functional MRI paradigm consisted of four blocks with a duration of 5:36 min each. The paradigm was performed in an ABBA versus BAAB block design with stimulation of either the clitoral region (A) or the right index finger (B) interspersed with 10-s periods of no stimulation, as previously described in Knop et al, 2022. Each of the four runs

	started with a period of no stimulation and included a total of eight clitoral and eight right index finger stimulation phases. The order of these phases was fixed and counterbalanced between women.
Behavioral performance measures	To monitor potential dislocation of the membrane during the procedure, women were asked to circle the placement of the membrane on an anatomical image of the genital region after the scan.

Acquisition

Imaging type(s)	Functional and structural (T1)
Field strength	3.0 T Siemens Magnetom Prisma fit scanner (Siemens Medical System) with a standard 64-channel head coil
Sequence & imaging parameters	One 0.8-mm3 isotropic T1-weighted anatomical scan was acquired using the magnetization-prepared rapid gradient echo sequence (MPRAGE) with a duration of 6:03 min and the following parameters TE=2.22 ms, TR=2,400 ms, flip angle=8, bandwidth=220 Hz/Px, FOV=256 mm.<br>Functional MRI scans were obtained using a T2*-weighted echoplanar image (EPI) pulse sequence (TR/TE = 2000/30 ms, slice number = 32, voxel size = 2 × 2 × 2 mm3, slice gap = 0.75 mm).<br>Further details on scanning parameters are available in the preregistered protocol (doi:10.17605/OSF.IO/QWEFS).
Area of acquisition	Whole-brain

Diffusion MRI ☐ Used ☒ Not used

Preprocessing

Preprocessing software	Anatomical preprocessing was carried out using the CAT12.9 (r2560) toolbox within SPM12, running on MATLAB_R2023b, with bias-corrected T1-weighted images. Functional preprocessing was performed using standard SPM12 functions. The first five “dummy” volumes were discarded to allow for T1 saturation. Double-pass realignment with default value and 4th degree interpolation was performed for the primary motion correction for additional precision. Intrasubject registration to the mean and anatomical image was separately performed for each of the four scanning blocks using default coregistration settings with anatomical image as a reference image and the mean image as a source image. Smoothing and normalization were purposefully omitted as the analysis was carried out in native space.
Normalization	Normalization was purposefully omitted, as the analysis was performed in native space.
Normalization template	See above.
Noise and artifact removal	All data was manually inspected for the presence of gross artifacts. For structural data, a quantitative quality metric was generated for each structural image in CAT12, and only images rated as "satisfactory" or higher (IQR > 70%) were included. For functional data, data was high-pass filtered with a default cutoff period of 128 s to correct for slow drift artifacts. Spin-echo field maps were acquired for distortion correction and applied via topup function in FSL.
Volume censoring	For structural data, T1-weighted images were manually reviewed to assess the presence of the tumors, aneurysms, white matter lesions or other clearly visible incidental findings. Images that were marked as suspicious were sent to a radiologist. In case of clinically relevant incidental findings, the subject’s data were excluded. For functional data, images were checked for motion parameters and images with absolute head motion above 3.0 mm and 3.0° of maximal translation and rotation in any direction throughout a scanning block were excluded.

Statistical modeling & inference

Model type and settings	Functional MRI data was analyzed using a general linear model (GLM) with two within-subject conditions of interest (10 s of either clitoral or right index finger stimulation alternating with 10 s of rest) modeled using a boxcar function convolved with a canonical hemodynamic response function (HRF).
Effect(s) tested	Activation maps were calculated with t-tests for contrasts between the two regressors of the design matrix: response to clitoral versus right index finger stimulation.

Specify type of analysis: ☐ Whole brain ☒ ROI-based ☐ Both

Anatomical location(s)	To localize the individual genital field, we saved the first most activated cluster in S1 as a region of interest. We then used the 10 most activated vertices identified through the above-described functional mapping paradigm as a region of interest. To further confirm that the identified region lies within S1, we normalized the saved region and checked it in Harvard–Oxford macroanatomical atlas available through JuBrain Anatomy Toolbox v3.0; Simon Eickhoff, Institut für Neurowissenschaften und Medizin, Forschungszentrum Jülich, Jülich, Germany
Statistic type for inference<br>(See Eklund et al. 2016)	Parametric inference using T-statistics, implemented in SPM12. Statistical significance was determined at the cluster level with a cluster-defining threshold of $p < 0.001$ (uncorrected) or $p < 0.05$ (FWE-corrected).
Correction	Since we only used the peak activation coordinate to identify the xyz coordinates for our region of interest to be used in a subsequent structural analysis and did not compare the groups on any of the functional parameters, we liberally thresholded

our functional data at $p < 0.001$ without correction or $p < 0.05$ with FWE-correction for multiple comparisons, as previously published in Knop et al., 2022.

Models & analysis

n/a	Involved in the study
☒	☐ Functional and/or effective connectivity
☒	☐ Graph analysis
☒	☐ Multivariate modeling or predictive analysis
